# The Unique Phenotype of Lipid-Laden Macrophages

**DOI:** 10.3390/ijms22084039

**Published:** 2021-04-14

**Authors:** Marco van Eijk, Johannes M. F. G. Aerts

**Affiliations:** Leiden Institute of Chemistry, Leiden University, 2333 CC Leiden, The Netherlands

**Keywords:** adipose tissue, foam cell, Gaucher disease, GPNMB, macrophage, multiple sclerosis, obesity, TREM-2

## Abstract

Macrophages are key multi-talented cells of the innate immune system and are equipped with receptors involved in damage and pathogen recognition with connected immune response guiding signaling systems. In addition, macrophages have various systems that are involved in the uptake of extracellular and intracellular cargo. The lysosomes in macrophages play a central role in the digestion of all sorts of macromolecules and the entry of nutrients to the cytosol, and, thus, the regulation of endocytic processes and autophagy. Simplistically viewed, two macrophage phenotype extremes exist. On one end of the spectrum, the classically activated pro-inflammatory M1 cells are present, and, on the other end, alternatively activated anti-inflammatory M2 cells. A unique macrophage population arises when lipid accumulation occurs, either caused by flaws in the catabolic machinery, which is observed in lysosomal storage disorders, or as a result of an acquired condition, which is found in multiple sclerosis, obesity, and cardiovascular disease. The accompanying overload causes a unique metabolic activation phenotype, which is discussed here, and, consequently, a unifying phenotype is proposed.

## 1. Introduction

Tissue macrophages are versatile cells involved, for example, in inflammatory responses and tissue repair. The activation of macrophages occurs via the recognition of pathogen-associated molecular patterns (PAMPs), damage-associated molecular patterns (DAMPs), and connected receptive signaling systems. Oversimplified, macrophage phenotypes can be divided into M1 (pro-inflammatory/classically activated) and M2 (anti-inflammatory/alternatively activated) subtypes. M1 activation occurs following recognition of the bacterial product lipopolysaccharide (LPS), interferon gamma (IFNγ), or a combination thereof. M2 activation can, for instance, occur upon stimulation with interleukin (IL)-4 or IL-10 [1,2,3]. This classic view touches the extremes of an array of phenotypes and a more detailed analysis revealed that a continuum and highly flexible pool of macrophage phenotypes can arise [4,5,6,7].

Due to their ingesting nature, macrophages are challenged with variable substrate loads during their life span and consequently are in need of a highly specialized compartment, the lysosome, to handle the burden [8]. Cargo enters the endo-lysosomal machinery via endocytosis (i.e., low-density lipoprotein particles with cholesterol), phagocytosis (i.e., pathogens), pinocytosis (fluid endocytosis), and autophagy (self-eating of worn-out organelles). Normally these uptake routes end by fusion events with lysosomes, the organelles wherein the actual catabolic machinery resides. Lysosomes are no longer viewed as catabolic endpoints solely involved in the degradation of macromolecules. The function of lysosomes shifted to a role as mediators of cell metabolism [9]. For instance, it is thought that lysosomes can sense the nutrient status of cells, control cell growth, division, and differentiation, are involved in immune responses, and terminate signaling cascades. Importantly, lysosomes can be induced transcriptionally on demand, for example, in a low nutrient state. Lysosomal dysfunction, either induced by defects in the catabolic machinery, or by too much lipid substrate entering for breakdown, perturbs cellular homeostasis. The latter has now been recognized to also drive the macrophage phenotype.

**Figure 1:**
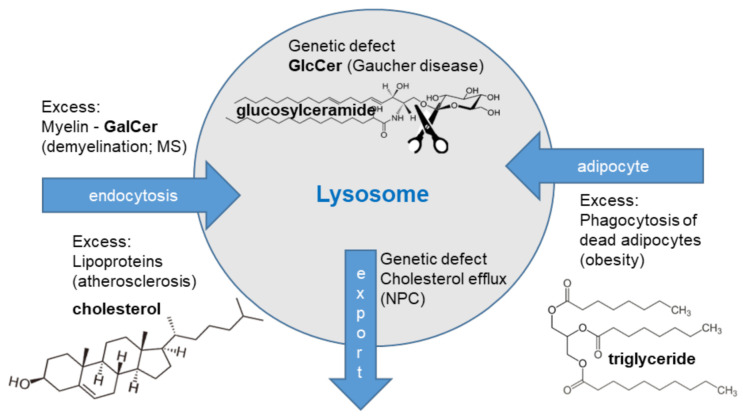
The lysosomal macrophage phenotypes. Gaucher disease is a genetic disease and glucosylceramide (GlcCer) accumulates within lysosomes of Gaucher cells. Niemann-Pick type C (NPC) is caused by a genetic defect in cholesterol transport proteins, driving cholesterol and glycosphingolipid accumulation in macrophages. Increased endocytosis of myelin causes increased lysosomal load in multiple sclerosis (MS) macrophages. Increased uptake of lipoprotein particles drives lysosomal lipid loading in atherosclerotic plaque macrophages. Uptake of apoptotic adipocytes causes triglyceride accumulation in adipose tissue macrophages.

In this review, we focus on the metabolically activated ‘lysosomal’ phenotype of macrophages. The paper discusses the occurrence of this macrophage phenotype when lysosome function is perturbed (Figure 1). Two different drivers of such a phenotype are also addressed.

First, the conditions characterized by flaws in the catabolic machinery are explained, exemplified by lysosomal storage disorders (LSD), Gaucher Disease (GD), and Niemann-Pick type C (NPC). Second, the acquired lipid overload condition, occurring, for instance, during multiple sclerosis (MS), obesity, and cardiovascular disease (CVD), are elaborated on. The ‘lysosomal’ phenotype and consequences of lipid overload in the context of macrophage-mediated inflammation are discussed.

## 2. The Lysosome

The lysosome was discovered in 1955 by Christian de Duve and co-workers by means of subcellular fractionation and the biochemical demonstration of membrane-enclosed acid hydrolase activities [10]. Soon after its recognition, this novel organelle was visualized by electron microscopy [11]. Lysosomes hold approximately 50 acid hydrolases, including proteases, lipases, nucleases, sulphatases, and glycosidases, involved in the catabolism of all sorts of macromolecules, allowing recycling of essential building blocks. Lysosomes are surrounded by a single lipid bilayer containing several lysosomal membrane proteins, including, among others, lysosome-associated membrane protein (LAMP)-1 and LAMP-2, lysosome integral membrane protein (LIMP)-2, and the tetraspanin CD63. Functionally, lysosomes contribute to metabolic homeostasis, plasma membrane repair, bone and tissue remodeling, immunity, cell death, and cell signaling. The vacuolar H^+^-ATPase (v-ATPase), an ATP-dependent proton pump, assures the acidic lysosomal lumen (pH 4.5–5.0), required for optimal catabolic activity [8,12].

### Regulation of Lysosome Biogenesis

**Figure 2:**
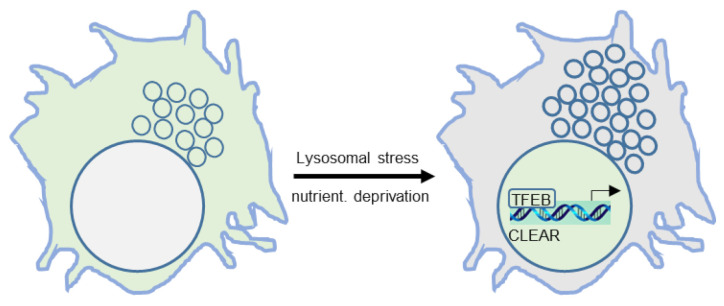
Transcriptional regulation of autophagy and lysosomal biogenesis, exemplified by transcription factor EB (TFEB) enriching in nuclei where it binds to the coordinated lysosomal expression and regulation (CLEAR) element in DNA, which is followed by transcription and translation.

The view that lysosomes are static end-stage catabolic household organelles that degrade macromolecules, which enter the lysosomes via endocytosis, phagocytosis, pinocytosis, or autophagy, has changed drastically after two key discoveries. First, it was shown that lysosomes are under the control of transcriptional regulation, with an important role for the transcription factor EB (TFEB), which binds to the coordinated lysosomal expression and regulation (CLEAR) motif in DNA (Figure 2). Consequently, a series of genes connected to lysosome biogenesis and autophagy are transcribed and translated [13,14].

Second, it was shown that the activity of the master growth-regulating kinase, a mechanistic target of rapamycin complex 1 (mTORC1), was connected to the nutrient status of cells. When plenty of nutrients (for example, amino acids) are around, cells can grow and mTORC1 is active and attached to the lysosome membrane. When nutrients are scarce, mTORC1 becomes inactive and detaches from lysosomes [15,16,17]. The nutrient status is sensed by the lysosomal nutrient-sensing complex (LYNUS). Importantly, the LYNUS machinery and transcriptional machinery turned out to be connected. Briefly, when nutrients are scarce, mTORC1 becomes inactive and this results in an altered phosphorylation state of TFEB, driving its nuclear localization and, as a consequence of binding to the CLEAR motif, induction of autophagy (bringing macromolecules to the lysosome for degradation) and lysosome biogenesis (the actual degrading machinery) [18]. Other microphthalmia-transcription factor E (MiT/TFE) family members, such as transcription factor E3 (TFE3), have also been shown to regulate lysosomal biogenesis [19]. Important to note here is that mTORC1 is not the only kinase connected to lysosomal biogenesis. In RAW 264.7 macrophage-like cells it was found that mTORC1 independent routes also exist. Lipopolysaccharide (LPS) and HEPES (cell culture media pH buffering agent) dependent transcriptional activation of lysosomal biogenesis both occurred via mechanisms differing from the starvation-induced mTORC1 route [20,21]. Recent research has revealed that at the surface of the lysosome a complex array of factors next to mTORC1 impact on the regulation of lysosome biogenesis and autophagy. The reader is referred to some excellent reviews on the topic [9,12,17].

## 3. The Catabolic Defective Storage Macrophages

### 3.1. Glycosphingolipid Metabolism

**Figure 3:**
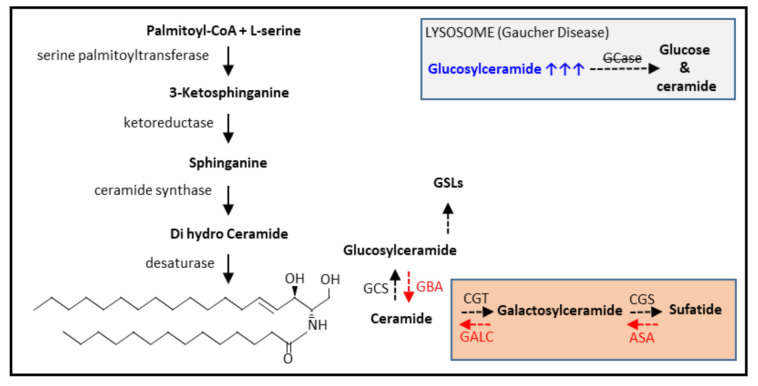
Brief representation of de novo ceramide synthesis and glycosphingolipid metabolism. GCS (glucosylceramide synthase), CGT (ceramide galactosyltransferase), CGS (cerebroside sulfotransferase). GBA (β-glucocerebrosidase/GCase), ASA (Arylsulfatase A), GALC (β-galactosylceramidase), GSLs (glycosphingolipids). In the grey box, Gaucher disease and glucosylceramide accumulation are depicted. In the orange box, the GSL species present in myelin are depicted. Black dashed arrows, GSL synthesis. Red dashed arrows, GSL breakdown.

To improve our understanding of how flaws in the lysosomal fragmentation of glycosphingolipids cause lysosomal distress and subsequent pathology, the biosynthesis of glycosphingolipids is first briefly described. The key sphingolipid that serves as a building block for all complex (glyco)sphingolipids (GSL) is ceramide and a simplified scheme is depicted in Figure 3. The formation of glycosylated ceramide species including galactosylceramide and its derivative sulfatide and glucosylceramide is included [22,23]. The degradation of GSL occurs within the lysosome by the stepwise removal of carbohydrate moieties. In the final degradation step, acid ceramidase splits the ceramide into free fatty acid and sphingosine. Genetic defects in breakdown enzymes cause GSL accumulation within lysosomes, which drives macrophage activation. During multiple sclerosis, phagocytes ingest a high load of myelin, which consists of, among other things, sulfatide and galactosylceramide and this will increase the lysosomal GSL load as well [24].

### 3.2. The Gaucher Cell

A genetic defect in lysosome function may lead to lysosomal distress, such as lipid accumulation. Macrophages are particularly prone to developing such accumulation given their role in the fragmentation of macromolecules. An example of this is offered by Gaucher cells in Gaucher Disease (GD) patients. These typical lipid-laden macrophages are found in subjects with GD throughout the body. For instance, these macrophages with tubular glucosylceramide deposits in their lysosomes accumulate in the bone marrow, spleen (especially red pulp macrophages involved in red blood cell clearance), liver, and lung. Gaucher cells arise as a consequence of mutations in the GBA gene, encoding the lysosomal enzyme acid β-glucocerebrosidase (GCase (EC 3.2.1.45); see grey inlay Figure 3), or, very rarely, defects in the PSAP gene, encoding the prosaposin protein, which among other things, can be processed into saposin C, an activator of GCase. In this most common LSD, the lack/impaired activity of lysosomal GCase results in the accumulation of glucosylceramide (GlcCer) within lysosomes [23,25]. This drives the specific macrophage phenotype, and, hence, the appearance of lipid-filled Gaucher cells. Early work already revealed that these cells produced high levels of a chitin degrading enzyme, named chitotriosidase [26]. To obtain further insights into the phenotype of human Gaucher cells, a detailed immunohistochemical analysis was performed. It was found that the cells resembled alternatively activated macrophages [27]. Macrophage origin was confirmed by CD68, CD14, and HLA class II positivity and the absence of dendritic cell markers. Inflammatory markers, such as tumor necrosis factor (TNF)-α, monocyte chemoattractant (MCP)-1, interleukin (IL)-1α, IL-12p40, and interferon-γ were not detected. Significantly, the cells surrounding the Gaucher cells do show inflammatory marker expression (MCP-1 and IL-1β). Interestingly, the anti-inflammatory markers chemokine (C-C motif) ligand 18 (CCL18), interleukin-1 receptor antagonist, and the scavenger receptor cysteine-rich type 1 protein M130 (CD163) were strongly expressed. In addition, the scavenger receptors scavenger/lipid receptor and signal-regulatory protein (SIRP)1α and CD36 were also expressed by Gaucher cells. Lysosomal acid phosphatase was highly positive as well extending on earlier differential splenic cDNA expression analysis revealing amongst others a lysosomal signature characterized by high expression of cathepsins B, K, and S, α-fucosidase, lysosomal acid lipase, and tartrate-resistant acid phosphatase (TRAP) [28]. A more recent LC-MSe-based proteome analysis of laser dissected Gaucher cells further extended the lysosomal phenotype exemplified by high protein levels of prosaposin and cathepsin D. This study also revealed a high induction of glycoprotein nonmetastatic melanoma protein B (GPNMB) [29]. As this protein is also shed, it also exists in a soluble form in the plasma of GD patients, showing potential as (macrophage-derived) biomarker. Importantly, in contrast to the existing human GD plasma markers chitotriosidase and CCL18, GPNMB is also present in rodent macrophages, which broadens its applicability in preclinical assessment [26,29,30,31]. GPNMB and chitotriosidase are also elevated in cerebrospinal fluid of GD patients, suggesting production by Gaucher cells present in the brain [32]. In addition, a tissue microarray analysis performed on the spleen and liver tissue of a GD mouse model points towards a lysosomal gene signature and inflammation. Based on the outcome, several potential biomarkers were suggested, including lysosomal enzymes, GPNMB, and CD9 [33]. Another study on GD mouse model tissues revealed a macrophage-enriched gene signature displaying a mixed M1/M2 nature and increased macrophage scavenger receptor-1 expression [34]. Of note, the inherited deficiency of acid sphingomyelinase causes Niemann-Pick diseases types A (NPA) and type B (NPB). NPA and NPB patients accumulate sphingomyelin-laden macrophages in the spleen and liver, so-called Pick cells, that show remarkable similarities in marker expression to Gaucher cells [35].

Summarizing, lipid-laden Gaucher cells express scavenger receptors, show a lysosomal gene signature, store GlcCer, are of a mixed classical/alternative activation phenotype, and express high levels of GPNMB.

### 3.3. The Niemann-Pick Type C Macrophage

The typical lipid-laden lysosomal storage macrophage phenotype is also observed in the LSD Niemann-Pick type C (NPC), which is caused by defects in intracellular cholesterol transporter proteins (NPC1/2). As a consequence of faulty cholesterol transport, the lysosomal hydrolase action becomes perturbed and this causes a secondary accumulation of various (glyco)sphingolipid species on top of non-esterified cholesterol deposition [36]. In mouse models of NPC, total spleen, brain, and liver gene expression profiles pointed to increased uptake (msr1, fabp5), more lysosomes (ctsb, ctss, ctsb, hexb, man2b1, and lyz1&2), lipid droplets (plin3, plin4), and innate immune activation, including macrophage involvement [37]. Several potential plasma detectable markers, including cathepsin S, cathepsin D, lysozyme, and galectin-3 have been proposed [37,38,39]. Lipid-laden NPC macrophages have not been carefully assessed regarding polarity as Gaucher cells. However, GPNMB is of interest in this respect. GPNMB showed a consistent induction in the spleen, brain, and liver of NCP mice [37,38]. This striking pattern mirrors the finding in Gaucher cells. Detailed histochemical analysis of the macrophages present in NPC mouse models confirmed the lipid-loaded appearance of Iba1^+^ cells in the spleen, brain, and liver. In line with the high GPNMB levels found in Gaucher cells, a detailed analysis of GPNMB also revealed high expression in these Iba1^+^ cells showing positive staining in red pulp macrophages (these macrophages undergo high lipid and iron pressure in the lysosomes due to erythrocyte turnover), splenic follicle center macrophages (these are involved in the removal of apoptotic germinal center B cells), Kupffer cells in the liver and in microglial cells in the brain [40]. In vitro studies using RAW264.7 cells, suggest that glucosylceramide, or a closely related lipid, drives GPNMB induction.

Concluding, lipid-filled NPC macrophages express scavenger receptors, show increased expression of lysosomal genes, droplet markers, low-grade inflammation, and a striking induction of GPNMB.

## 4. The Acquired Storage Macrophages

The aforementioned lysosomal phenotype arises due to a genetic defect. In the following section, several acquired lysosomal macrophage phenotypes are discussed. This phenotype arises when a macrophage either acutely, or for a prolonged period, encounters an increased load of lipid substrate that needs to be handled (Figure 1). This challenging environment puts the lysosomal capacity to its limit, or even over its limit, driving a LSD-like macrophage phenotype. Three examples of such macrophages in (acquired) diseases are discussed, namely, the myelin-laden multiple sclerosis macrophage, the obese lipid-loaded metabolic activated (MMe) adipose tissue macrophage, and the cholesterol-filled foam cell observed during CVD.

### 4.1. Multiple Sclerosis

Multiple sclerosis (MS) is a neurological disorder of the central nervous system. The neuroinflammatory environment is a result of axonal loss, which is attributed to demyelination events [41,42]. Myelin offers physical and trophic support to neurons and aids the speed of action potential conduction along axonal fibers [43]. During MS-activated microglia, recruited monocyte-derived macrophages contribute to demyelination [44]. Myelin is rich in lipids and contains high levels of cholesterol and is enriched in glycosphingolipids (for instance, galactosylceramide and sulfatide, see Figure 3). During demyelination, it is obvious that the lysosomes of the myelin engulfing macrophages encounter a highly increased load of lipids to digest, pushing the catabolic machinery to, or over, their limit. This challenging environment may cause a (temporary) lysosomal storage phenotype. In agreement, foamy macrophages are encountered all overactive MS lesions, whereas in chronic lesions a demyelinated sclerotic core contains encircling foamy cells [45,46]. Immunohistochemical analysis using several criteria, namely HLA-DR positivity, myelin oligodendrocyte protein (MOG) positivity, and the presence of neutral lipids using oil red O staining and size revealed different foam cell populations. Foam cells were present within the lesion, in perivascular spaces within the lesion and in the outer (smaller, more MOG), or inner rim. The expression of pro-inflammatory cytokines TNF-α, IL-1β, and IL-12p40/70 was not detected in any of the foam cells. Anti-inflammatory molecules IL-1ra, CCL18, IL-10 (exception is the perivascular foam cell), TGF-β, and IL-4 were all expressed by foamy macrophages, most prominently in the center of the lesion. The foamy macrophages resemble anti-inflammatory macrophages and display similarity with Gaucher cells. The foam cell phenotype could be recapitulated in monocyte-derived macrophages by loading with human brain-derived myelin and these macrophages were also of an immunosuppressive nature [47]. Foamy macrophages also expressed high levels of scavenger receptors, including SR-AI/II, CXCL16, LOX-1, and LRP-1 [48]. In addition, transcriptional profiling demonstrated the induction of Gaucher cell-associated markers, such as chitotriosidase, GPNMB, and CCL18 [49]. The analysis of the transcriptional profile of isolated human microglia from normal-appearing white matter from MS patients revealed genes associated with foam cell differentiation, lipid catabolism (lysosomes, LPL), and storage. GPNMB was highly induced as well. It was speculated that peroxisome proliferator-activated receptor (PPAR)γ activation may drive an anti-inflammatory signature [50]. Importantly, the triggering receptor expressed on myeloid cells (TREM)-2 turned out to be driving the formation of disease-associated microglia (DAM) [51,52]. TREM-2 caused the accumulation of pathogenic lipid species in microglia during MS. In addition, TREM-2 regulated the lysosomal and lipid metabolism signature and correlated well with CD9 [52].

In conclusion, myelin-laden macrophages in MS are equipped to take up myelin and display a lysosomal and lipid metabolism (uptake and storage) signature and a suppressed inflammation phenotype and are highly GPNMB positive.

### 4.2. Obesity

Obesity is increasing at an alarming rate and is accompanied by an increased risk to develop insulin resistance, type 2 diabetes, cardiovascular disease, and cancer for instance [53]. The imbalance between energy consumed and energy burnt causes adipose tissue (AT) to evolve. During obesity, adipocytes increase in number and undergo hypertrophy and metabolic changes [54]. This is also accompanied by low-grade inflammation, and, hence, the name metabolic inflammation (met-inflammation) arose [55,56]. One of the first observations connecting AT inflammation to insulin resistance and obesity dates from studies on tumor necrosis factor (TNF)-α and efficacy of anti-TNF-α treatment in obese rodents [57]. A decade later 2 key papers revealed that macrophages are essential components of inflamed obese AT [58,59]. Lean AT contains approximately 10% macrophages, which increases up to 40% during obesity, and the macrophages show a foamy appearance [58,59]. The peculiar appearance of AT macrophages (ATM) in obese tissue led to the name crown-like structures (CLS), and, hence, the macrophages surrounding dead adipocytes [60]. Further characterization of macrophage populations in AT disclosed that ATM undergo a phenotype switch from an alternatively activated phenotype (lean AT) to an inflammatory phenotype (obese AT) [61]. Relevant in the context of this review is the phenotype reported by scientists from Cambridge, who noted a differential lipid distribution during obesity from adipocytes to macrophages. It was demonstrated that, during obesity, the foam cells arise after an initial M2 phase. This foamy ATM population started expressing high levels of CD36 and fatty acid transporter protein (FATP)-1 favoring lipid uptake and displayed high expression of lipoproteinlipase (LPL) to handle the lipids [62]. The accumulating triglycerides predominantly contained saturated short-chain fatty acids. Thus, it seems that the macrophage provides a lipid sink when the adipocyte fails to handle the lipids. A similar hypothesis was postulated earlier in a study in which the lowering of glycosphingolipids improved adipocyte function, consequently reducing lipid pressure on ATM [63]. A decade after the initial documentation of increased macrophage content in obese AT, it was found that obese ATM showed an increase in lysosomal content accompanied by lipid metabolism pathways [64]. Lysosomes were visualized in obese ATM by electron microscopy and by lysotracker staining. Increased body mass correlated well with genes encoding lysosomal proteins including cathepsins, acid lipase A, lysosomal-associated membrane protein-2, and NPC-1. Importantly, the differentiation of macrophages in the presence of AT induced lysosomal biogenesis (Atp6v0d2, Lipa, and Ctsk) without inducing an overt pro-inflammatory phenotype (no induction of tnf and tlr2 and tlr4), but induction of Arg1, Il1b, and Nos2 was observed. Again, a lipid storage prone signature was found, including the induction of scavenger receptors (Msr1) and storage proteins (Plin2). The protein GPNMB already discussed in GD, NPC, and MS storage macrophages is also induced in obese ATM. Strong GPNMB immunostaining was observed in obese AT CLS and, in agreement, FACS sorted obese ATM populations showed high gene expression [65]. In vitro, several lysosomal stressors, including chloroquine, concanamycin A, bafilomycin A1, and lipid loading (palmitate, but not oleate) also induced GPNMB. The discussed lipid-accumulating macrophages, occurring in GD, NPC, and MS, all seem to deviate from the pro-inflammatory classically activated versus the anti-inflammatory alternatively activated paradigm. Oversimplified, it seems that storage macrophages, in general, are equipped with scavenger receptor systems promoting the uptake of lipid cargo, machinery to process (lysosomes) and store (lipid droplets), and displaying a mixed inflammatory phenotype. Interestingly, this phenotype resembles the macrophage phenotype arising after ‘metabolic activation’ (MMe) with high levels of glucose, insulin, and palmitate [66]. The MMe differs from M1 and M2 macrophages. MMe express ABCA1, CD36 and PLIN2, and IL1β and TNFα. The cytokine levels are lower when compared to classically activated macrophages. Both PPARγ activation and p62 accumulation, which is the consequence of autophagy inhibition, are implied in this regulation. In addition, MMe do not mount a type I interferon response, as IRF7 is not induced. Functionally, the MMe are both detrimental (production of inflammatory mediators) and beneficial (via lysosomal excretion dead adipocytes are cleared and ectopic lipid accumulation is avoided) [67]. The earlier key findings on ATM subpopulations have now been extended by using single-cell RNA sequence analysis based strategies. This has resulted in the discovery of additional ATM subsets. Hill and colleagues demonstrated three obese ATM populations, based on CD9 and Ly6c^−^ expression [68]. Importantly, the CD9^+^ ATM population accumulates in CLS. This ATM population is lipid-laden and lysosome-enriched, as demonstrated by BODIPY staining and gene expression profiles (Acp5, cts, Lpl, Plin2, Lamp1, Lamp2, Npc1, Npc2 etc.). In line with the high expression of the tetraspandin CD9 and its earlier functional connection to exosomes, this ATM population also secretes exosomes. The inflammatory signature again is not a clear cut M1, or M2 signature, but inflammatory markers are present. The existence of this CD9^+^ lipid accumulating ATM was also confirmed in human obese tissue CLS and CD9^+^ ATM number correlated well with BMI. Another time-resolved unbiased single-cell RNA sequencing study also found a gene signature favoring phago/endocytosis, lysosomes, and lipid metabolism. In a subset of lipid-associated macrophages (LAM) high gene expression of Trem2, Lipa, Lpl, Ctsb, Ctsl, Fabp4, Fabp5, Lgals1, Lgals3, Cd9, Cd36, and GPNMB was found. Functionally, it was demonstrated that TREM-2, acting as a lipid receptor, steered foam cell development and the MMe phenotype [69]. The signature was also recapitulated in human obese AT. Human TREM-2^+^ LAM cells displayed a gene signature (LIPA, CTSB, CTSL, FABP4, FABP5, LGALS3, CD9, and CD36) comparable to LAM in mice. Importantly, TREM-2 is also implied in other lipid accumulating macrophage-associated diseases. The important role of lysosomes in ATM was further supported by a study in which transgenic overexpression of TFEB in macrophages triggered lysosomal biogenesis, which protected mice against HFD-induced obesity and insulin resistance with a key role for growth differentiation factor 15 (GDF15) [70].

Overall, obese AT foam cells are prone to take up lipid cargo, contain lysosomes to degrade, and lipid droplets to store material, and display a mildly inflammatory phenotype and express GPNMB.

### 4.3. Cardiovascular Disease

CVD is the number one cause of death worldwide. Approximately 17.9 million people die from CVD annually, which corresponds to 31% of all deaths, as reported by the World Health Organization. CVD comprises a group of heart and blood vessel disorders including coronary heart and cerebrovascular disease, giving rise to either heart attacks, or strokes. The cause of these diseases lies in the gradual accumulation of lipids, the development of inflammation, and ultimately the formation of fibrotic tissue in the inner lining of the arterial wall. In the end, blood flow obstruction arises, or becomes insufficient, causing the tissue to perish. Alternatively, plaque rupture may occur driving detrimental thrombus formation [71,72]. Foam cells are key in developing atherosclerotic plaques. The uptake of oxidized low-density lipoprotein (oxLDL) particles triggers the formation of foam cells and inflammatory response. Recruited monocytes mature into macrophages and initially attempt to remove the local overshoot of toxic lipids. Atherosclerotic plaque foam cells express various lipid scavengers, including scavenger receptor (SR)-A, CD36, and lectin-like LDL receptor (LOX)-1, involved in the uptake of modified LDL. After uptake, cholesterol is esterified and further metabolized into free fatty acid and cholesterol and the latter is exported by ATP-binding cassette transporters and SR-B1 [73]. This process, however, becomes perturbed, partly due to the fact that oxLDL uptake intervenes with lysosomal enzyme localization and activity, resulting in additional lipid accumulation, and, hence, more foam cell formation [74]. The oxLDL matured foam cells do not fit the M1 or M2 categorization, but rather resemble the previously described MMe phenotype. In the 70s, De Duve already stated that foam cell formation was a lysosomal storage disease [75]. This view was further extended by the demonstration of chitotriosidase (discovered in GD) and TRAP (also connected to GD), in atherosclerotic lesion macrophage subsets [26,28,76]. Taken together, foam cells in advanced atherosclerotic lesions displayed an acquired lysosomal storage phenotype [77,78]. Importantly, it has been demonstrated that macrophage-specific expression of TFEB, which induces lysosomal biogenesis, is antiatherogenic [79,80]. Again, recent technological progress, and, hence, the use of single-cell RNA SEQ analysis, boosted insights into macrophage populations and markers in plaques [81,82,83,84,85]. Three different macrophage populations have been proposed to exist in atherosclerotic aortas, namely, resident-like macrophages, inflammatory macrophages, and TREM-2^hi^ foamy macrophages [81]. The latter two being more associated with atherosclerosis. The TREM-2^hi^ cells also express CD9, lysosomal genes, GPNMB, galectin-3, and osteopontin. This phenotype supports ingestion, catabolism, lipid metabolism, regulation of cholesterol efflux, and oxidative stress. The foam cells are not clearly fitting the M1 or M2 signature. Together, the TREM-2^hi^ cells demonstrate a clear resemblance with the foamy MMe phenotype arising during obesity and lipid accumulating microglia in MS [52,66,81,82]. Comparable analysis using human atherosclerotic plaques recapitulated the phenotype, including TREM-2, GPNMB, and CD9, in general supporting lipid uptake, lipid metabolism, and lipid storage and displaying a suppressed inflammatory phenotype [84,86].

Thus the atherosclerotic plaque foam cell scavenges lipids, catabolizes (lysosomes) and stores (droplet protein) the lipids, shows a suppressed inflammatory phenotype, and is GPNMB positive.

## 5. In Vitro Models

As discussed, various in vitro lipid-laden cell models exist, including the generation of Gaucher cells (of note, glucosylceramide levels do not reach anywhere near the levels observed in vivo), MS foam cells (myelin-laden monocyte-derived macrophages), MMe (high glucose, insulin, and palmitate), an atherosclerotic plaque foam cell (ox-LDL feeding) [47,66,73,87,88]. To mimic lysosomal perturbations other interventions have also been employed. Macrophages can be cultured in the presence of compounds triggering an increase of their lysosomal pH, such as chloroquine (a weak lysosomotropic base acting as H^+^ sponge), or vacuolar H^+^-ATPase inhibitors such as bafilomycin A1, or concanamycin A. Sucrose feeding to cells also induces a storage cell, due to the absence of the catabolic machinery (only the small intestine is equipped with sucrase). As discussed earlier, a tight connection exists between nutrient status and mTORC1 activity. Using mTORC1 inhibitors such as Torin-1, lysosomal pathways can also be boosted. Lastly, the frequently used cell culture buffering agent HEPES influences the lysosomal compartment. HEPES (25mM, the commercially present concentration) addition to cell culture media induces cytosol to nucleus movement of the microphthalmia-transcription factor E (MiT/TFE) members TFEB, TFE3, and MITF and causes lysosomal biogenesis. RNA seq analysis performed on ‘HEPES’ RAW264.7 cells revealed a unique signature when compared to classically activated (LPS), or alternatively activated (IL-4) stimulated cells, characterized by a strong lysosomal gene signature, but modest inflammation when compared to LPS. Furthermore, a very robust induction of GPNMB occurred in the cells [21,89]. Thus, the use of HEPES in certain cell culture experiments and lysosomal enzyme diagnostics should be avoided, or at least the effects should be taken into consideration. Thus, several models exist to address lysosomal perturbations in macrophages.

## 6. Summary and Future Perspectives

**Figure 4:**
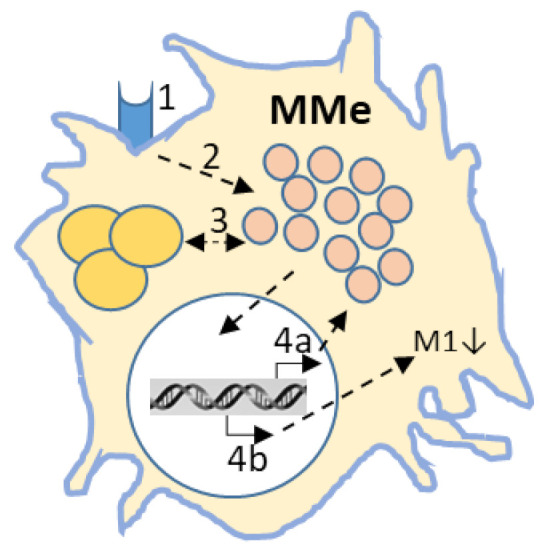
The lipid-laden macrophage. 1. Lipid cargo enters macrophages through uptake by scavenger receptors (CD36, TREM-2), or via phagocytosis of red blood cells, or apoptotic cells. 2. Captured lipids will be broken down in lysosomes. Due to defects in the catabolic machinery (GD, or NPC), or due to acquired overload (myelin, TG, or oxLDL) lipid material will be stored in 3. droplets/lysosome. 4. Transcriptional regulation a. MiT-TFE driven lysosomal biogenesis and autophagy and b. M1 phenotype suppression (LXR/PPARγ). Induction of p62, occurring as a consequence of autophagy inhibition also contributes to immune suppression.

There are similarities between lipid-laden macrophages, both those stemming from genetic defects, such as deficiencies in lysosomal proteins (e.g., in GD and NPC) and those caused by an excessive lipid load (myelin in MS, TG during obesity and cholesterol in atherosclerosis). This is summarized in Figure 4. The accumulation of lipids in lysosomes of macrophages significantly impacts their metabolism, but in a different way to classical (M1) and alternative activation (M2). Glycolysis is strongly induced in M1 macrophages, supporting inflammation. Oxidative phosphorylation is strongly induced in IL-4 matured M2 macrophages [90]. Obese ATM show a unique metabolic activation profile [91]. In addition, the epigenetic landscapes are different between M1, M2, and atherosclerosis foam cells [92]. It has been postulated that the repressed inflammatory phenotype in MMe is a consequence of PPARγ, or alternatively by LXR activation, and p62 induction [66,93]. In other words, the MMe phenotype is unique.

The recently increased insight regarding TREM-2 driving the MMe phenotype warrants discussion. TREM-2 is present on lipid-laden macrophages in the obese adipose tissue (LAM), lipid-laden phagocytes in MS (DAM), and foam cells in atherosclerotic plaque [52,69,81]. TREM-2 loss of function mice do not form the foamy LAM population during obesity and this is accompanied by worsening of glucose homeostasis [69]. It has been speculated that contributing to this are disturbed ceramides, a class of lipids implicated in insulin resistance [94,95]. The LAM transcriptome signature resembles that of DAM [51]. In the absence of TREM-2, the DAM in MS fail to induce the gene program to optimally handle the lipid load, and, consequently, unfavorable cholesterol ester accumulation occurs [52,96]. Foam cells present in atherosclerotic plaques also express TREM-2 and it is highly likely that this contributes to the lipid handling signature in CVD as well [81]. It, thus, seems that TREM-2 is key for igniting the program to deal with lipid overload to prevent lipotoxicity of the macrophages, and also other cells and tissues. Indeed, TREM-2 activation on microglia has been shown to aid myelin clearance and boost remyelination in mice treated with the copper chelator cuprizone [97]. Of note, TREM-2 activation also is connected to the development of a suppressive tumor environment [98].

Another striking finding is the strong induction of GPNMB in all types of lipid-laden macrophages. A soluble GPNMB fragment is shed from cells and its presence in plasma is used to monitor disease in some LSDs [29,40,89]. Of note, GPNMB is reported to suppress T cell activation (via syndecan-4), to promote M2 polarization, and tissue repair, and is hypothesized to be involved in the trafficking of phagocytosed debris and the fusion of autophagosomes and lysosomes [99,100,101]. Although the precise function of GPNMB is not yet fully understood, it is possibly involved in coping with excessive lipid stress as suggested by its prominent presence in lipid-laden macrophages.

CD9, a tetraspanin family protein, is also expressed on TREM-2^hi^ macrophages. Interestingly, CD9^+^ adipose macrophages secrete exosomes [68]. The CD9^+^ miRNA containing exosomes, influences (positively in lean -and negatively in obese mice) insulin sensitivity [102]. A more detailed analysis of the ATM-derived CD9^+^ exosomes, i.e., assessment of both protein and lipid composition, may uncover their intriguing function.

The remarkable observed similarities between lipid-laden macrophages in the inherited LSD and those acquired in MS and during obesity and atherosclerosis are intriguing. It can be envisioned that the ongoing development of therapies for LSDs might ultimately also provide therapeutic avenues for common acquired diseases involving lipid-laden macrophages. Vice versa, further knowledge on DAM may provide insights into the neuropathology that is commonly developing in LSDs.
